# Author Correction: The value of ecosystem services in global marine kelp forests

**DOI:** 10.1038/s41467-023-38666-4

**Published:** 2023-05-18

**Authors:** Aaron M. Eger, Ezequiel M. Marzinelli, Rodrigo Beas-Luna, Caitlin O. Blain, Laura K. Blamey, Jarrett E. K. Byrnes, Paul E. Carnell, Chang Geun Choi, Margot Hessing-Lewis, Kwang Young Kim, Naoki H. Kumagai, Julio Lorda, Pippa Moore, Yohei Nakamura, Alejandro Pérez-Matus, Ondine Pontier, Dan Smale, Peter D. Steinberg, Adriana Vergés

**Affiliations:** 1 School of Biological, Earth, and Environmental Sciences, University of New South Wales, Sydney, NSW 2033 Australia; 2 Kelp Forest Alliance, Sydney, NSW 2034 Australia; 3 The University of Sydney, School of Life and Environmental Sciences, Sydney, NSW Australia; 4 Singapore Centre for Environmental Life Sciences Engineering, Nanyang Technological University, Singapore, Singapore; 5 Sydney Institute of Marine Science, Mosman, NSW Australia; 6 Universidad Autónoma de Baja California, Facultad de Ciencias Marinas, Ensenada, BC Mexico; 7 Leigh Marine Laboratory, Institute of Marine Science, University of Auckland, Auckland, New Zealand; 8 Commonwealth Scientific and Industrial Research Organization, Environment, Brisbane, QLD 4072 Australia; 9 Department of Biology, University of Massachusetts Boston, Boston, MA 20125 USA; 10 School of Life and Environmental Sciences, Deakin University, Queenscliff, VIC 3225 Australia; 11 Department of Ecological Engineering, Pukyong National University, Busan, South Korea; 12 Hakai Institute, Quadra Island, Canada; 13 Institute of the Oceans and Fisheries, University of British Columbia. 2202 Main Mall, Vancouver, BC V6T 1Z4 Canada; 14 Department of Oceanography, College of Natural Sciences, Chonnam National University, Gwangju, 61186 Korea; 15 Center for Climate Change Adaptation, National Institute for Environmental Studies, 16-2 Onogawa, Tsukuba, Ibaraki 305-8506 Japan; 16 Universidad Autónoma de Baja California, Facultad de Ciencias, Ensenada, BC, Mexico & The Tijuana River National Estuarine Research Reserve, Imperial Beach, CA USA; 17 School of Life Sciences, Aberystwyth University, Aberystwyth, SY23 3DA UK; 18 Dove Marine Laboratory, School of Natural and Environmental Sciences, Newcastle University, Newcastle-upon-Tyne, NE1 7RU UK; 19 Graduate School of Integrated Arts and Sciences, Kochi University, 200 Monobe, Nankoku, Kochi 783-8502 Japan; 20 Subtidal Ecology Laboratory (Subelab), Estación Costera de Investigaciones Marinas (ECIM), Departamento de Ecología, Facultad de Ciencias Biológicas, Pontificia Universidad Católica de Chile, Casilla, 114-D Santiago Chile; 21 Millennium Nucleus for the Ecology and Conservation of Temperate Mesophotic Reef Ecosystem (NUTME), Las Cruces, Valparaiso Chile; 22 Marine Biological Association of the United Kingdom, Citadel Hill, Plymouth PL1 2PB UK

**Keywords:** Environmental economics, Biodiversity, Marine biology

Correction to: *Nature Communications* 10.1038/s41467-023-37385-0, published online 18 April 2023

The original version of this article omitted a reference to previous work in ‘Eger et al. 2022 - Eger, A. M, Bennett, S., Zimmerhackel, J., Rogers, A., Burton, M., Filbee-Dexter, K., Wernberg, T., Gacutan, J., Milligan, B., Vergés, A. (2022) Quantifying the ecosystem services of the Great Southern Reef. Report to the National Environmental Science Program. University of New South Wales. Available at: https://www.nespmarinecoastal.edu.au/project-1-9-final-report-2/’and ‘Zimmerhackel et al. 2023 - Zimmerhackel, J. S., Pineiro-Corbeira, C., Norderhaug, K. M., Filbee-Dexter, K., & Wernberg, T. (2023) Dependency of commercial fisheries on kelp forests for valuation of ecosystem services. Working Paper 2302, Agricultural and Resource Economics, The University of Western Australia, Crawley, Australia. 10.22004/ag.econ.334183’.

These have been added as references 110, 111 at: ‘We accounted for this fact by creating dependency classes for 187 genera of fish and invertebrates, adapted from refs. ^110,111’^. These references have been added to the reference list.

The original version of this article contained an error in the “Acknowledgements”, which incorrectly omitted the following: ‘A.E., A.V., D.S., and P.M. would also like to acknowledge our continued collaboration with the GEAK network and our valued discussions on how to value ecosystem services in kelp forests. In particular, we thank Johanna Zimmerhackel, Cristina Pineiro-Corbeira, Kjell-Magnus Norderhaug, Karen Filbee-Dexter, and Thomas Wernberg for their thinking and work on fisheries dependencies in kelp forest ecosystems.’

This has been corrected in both the PDF and HTML versions of the article.

