# Supplementary material for: The value of ecosystem services in global marine kelp forests
Source: Nat Commun. 2023 Apr 18;14:1894. doi: 10.1038/s41467-023-37385-0 (PMC10113392; doi:10.1038/s41467-023-37385-0)
Supplement: Supplementary file 2 — Description of Additional Supplementary Files [file 41467_2023_37385_MOESM2_ESM.pdf]

Description of Additional Supplementary Files:

Supplementary Datasets 1-14: Datasets and values used to calculate the values presented in the study.

Supplementary Data 1: Nutrient content and NPP measurements

Supplementary Data 2: Site level fisheries values

Supplementary Data 3: Sensitivity analysis for different fisheries extraction rates and the resulting economic value per hectare per year

Supplementary Data 4: Projected carbon capture potential of kelp genera

Supplementary Data 5: Kelp distribution data

Supplementary Data 6: Classifications for genera dependency on kelp forest habitat

Supplementary Data 7: Sensitivity analysis for carbon sequestration

Supplementary Data 8: Search terms

Supplementary Data 9: Biodiversity Surveys

Supplementary Data 10: Weight-length coefficients and weights of species

Supplementary Data 11: Fisheries cost data

Supplementary Data 12: Cost of capital by country

Supplementary Data 13: Discount rates used for adjusting different processing and risk factors associated with market prices

Supplementary Data 14: Costs of nutrient schemes
