# Supplementary material for: The value of ecosystem services in global marine kelp forests
Source: Nat Commun. 2023 Apr 18;14:1894. doi: 10.1038/s41467-023-37385-0 (PMC10113392; doi:10.1038/s41467-023-37385-0)
Supplement: Supplementary file 4 — Reporting Summary [file 41467_2023_37385_MOESM4_ESM.pdf]

nature portfolio

Corresponding author(s): Aaron Eger

Last updated by author(s): Feb 27, 2023

Reporting Summary

Nature Portfolio wishes to improve the reproducibility of the work that we publish. This form provides structure for consistency and transparency in reporting. For further information on Nature Portfolio policies, see our Editorial Policies and the Editorial Policy Checklist.

Statistics

For all statistical analyses, confirm that the following items are present in the figure legend, table legend, main text, or Methods section.

n/a	Confirmed	
[ ]	[x]	The exact sample size (*n*) for each experimental group/condition, given as a discrete number and unit of measurement
[x]	[ ]	A statement on whether measurements were taken from distinct samples or whether the same sample was measured repeatedly
[x]	[ ]	The statistical test(s) used AND whether they are one- or two-sided<br>*Only common tests should be described solely by name; describe more complex techniques in the Methods section.*
[x]	[ ]	A description of all covariates tested
[x]	[ ]	A description of any assumptions or corrections, such as tests of normality and adjustment for multiple comparisons
[ ]	[x]	A full description of the statistical parameters including central tendency (e.g. means) or other basic estimates (e.g. regression coefficient) AND variation (e.g. standard deviation) or associated estimates of uncertainty (e.g. confidence intervals)
[x]	[ ]	For null hypothesis testing, the test statistic (e.g. *F*, *t*, *r*) with confidence intervals, effect sizes, degrees of freedom and *P* value noted<br>*Give P values as exact values whenever suitable.*
[x]	[ ]	For Bayesian analysis, information on the choice of priors and Markov chain Monte Carlo settings
[x]	[ ]	For hierarchical and complex designs, identification of the appropriate level for tests and full reporting of outcomes
[x]	[ ]	Estimates of effect sizes (e.g. Cohen's *d*, Pearson's *r*), indicating how they were calculated

Our web collection on statistics for biologists contains articles on many of the points above.

Software and code

Policy information about availability of computer code

Data collection	No software were used in the data collection process.
Data analysis	All data were analyzed using the R programming language V4.0.0 and R Studio V1.4.1717.<br><br>All the code required to achieve the project outputs is located at osf.io/ykqc3

For manuscripts utilizing custom algorithms or software that are central to the research but not yet described in published literature, software must be made available to editors and reviewers. We strongly encourage code deposition in a community repository (e.g. GitHub). See the Nature Portfolio guidelines for submitting code & software for further information.

Data

Policy information about availability of data

All manuscripts must include a data availability statement. This statement should provide the following information, where applicable:

- Accession codes, unique identifiers, or web links for publicly available datasets
- A description of any restrictions on data availability
- For clinical datasets or third party data, please ensure that the statement adheres to our policy

All data related to this study are uploaded to the Open Science Framework and are available at osf.io/ykqc3

Human research participants

Policy information about studies involving human research participants and Sex and Gender in Research.

Reporting on sex and gender	*Use the terms sex (biological attribute) and gender (shaped by social and cultural circumstances) carefully in order to avoid confusing both terms. Indicate if findings apply to only one sex or gender; describe whether sex and gender were considered in study design whether sex and/or gender was determined based on self-reporting or assigned and methods used. Provide in the source data disaggregated sex and gender data where this information has been collected, and consent has been obtained for sharing of individual-level data; provide overall numbers in this Reporting Summary. Please state if this information has not been collected. Report sex- and gender-based analyses where performed, justify reasons for lack of sex- and gender-based analysis.*
Population characteristics	*Describe the covariate-relevant population characteristics of the human research participants (e.g. age, genotypic information, past and current diagnosis and treatment categories). If you filled out the behavioural & social sciences study design questions and have nothing to add here, write "See above."*
Recruitment	*Describe how participants were recruited. Outline any potential self-selection bias or other biases that may be present and how these are likely to impact results.*
Ethics oversight	*Identify the organization(s) that approved the study protocol.*

Note that full information on the approval of the study protocol must also be provided in the manuscript.

Field-specific reporting

Please select the one below that is the best fit for your research. If you are not sure, read the appropriate sections before making your selection.

☐ Life sciences ☐ Behavioural & social sciences ☒ Ecological, evolutionary & environmental sciences

For a reference copy of the document with all sections, see nature.com/documents/nr-reporting-summary-flat.pdf

Ecological, evolutionary & environmental sciences study design

All studies must disclose on these points even when the disclosure is negative.

Study description	We compiled 3 datasets, 1 that describes three observed ecosystem services provided by 4 genera of marine ecosystem engineers (here in, kelp), 1 that attributed economic values to the services provided, and 1 that estimated the areal coverage of those kelp ecosystems.<br><br>These data were then used to calculate the area adjusted measure and economic value of kelp ecosystem services per year as well as the absolute economic value of kelp ecosystem services across the globe.
Research sample	The research sample represented the available information for three ecosystem services provided by kelp forests<br><br>1) Fisheries production<br>2) Nutrient removal<br>3) Carbon capture<br><br>The kelp genera were selected because they are the most widely distributed kelp species across the globe and cover the approximate range occupied by all species within that order (Laminariales).<br><br>We selected the three services because they are viewed as the most important services and they have well established market values that could be sourced and given an attributed dollar value.
Sampling strategy	We conducted comprehensive literature reviews and combined those findings with privately held datasets. We also worked with researchers in countries that publish their work in languages other than English. As such, we aimed to collect as much of the available information as possible. Inevitably, we have not captured every potential data point but have produced a robust dataset based on the available information.
Data collection	The primary author conducted the literature searches for the fisheries biodiversity surveys, kelp net primary production rates, kelp elemental composition, and coordinated the collection of the unpublished datasets related to these same services. The published data were collected from the papers while the unpublished data was compiled by the primary author into a single datasheet.<br><br>Undergraduate researchers assisted in searching for market values of fishery species as well as the weight-length coefficients required to calculate the fish biomass. These values were then independently verified by a different undergraduate researcher.<br><br>The primary author collected the market prices for nutrient removal and carbon capture. All available information was collected for

	nutrient removal while a commonly used value (the social price of carbon) was used for carbon capture.
Timing and spatial scale	The data were collected from all locations and time points, there were no exclusions based on time and location.<br><br>The literature searches for fishery biodiversity surveys, kelp net primary production, and kelp element composition, were conducted from March 2020 to May 2020.<br><br>The solicitation of unpublished datasets was conducted from April 2020 to January 2022.<br><br>The fishery market values and fish weight-length coefficient searches were conducted from April 2020 to December 2021.<br><br>The nutrient and carbon market value searches were conducted in April 2020 and reviewed in November 2021.
Data exclusions	There were no exclusions to data, all available information was collected.
Reproducibility	We recorded the search terms, engines, dates, and outputs used to conduct the literature searches. We verified that the published data searches are reproducible.<br><br>We also recorded all data sources as references or URLs and the data is therefore traceable and could be collected again with the same results. Some price data may have changed as the market prices fluctuate but we noted the time of collection to allow for the data to be pulled from archived website. We have verified that the URLs were correct at the time of submission.
Randomization	We could not randomize the data collection because this work aimed to synthesize all available data and controls and randomization would not be appropriate for a synthetic literature analysis.
Blinding	As above, we aimed to collect all the data that existed on the subject and thus there was no need to assign groups for data collection or otherwise.

Did the study involve field work? ☐ Yes ☒ No

Reporting for specific materials, systems and methods

We require information from authors about some types of materials, experimental systems and methods used in many studies. Here, indicate whether each material, system or method listed is relevant to your study. If you are not sure if a list item applies to your research, read the appropriate section before selecting a response.

Materials & experimental systems

n/a	Involved in the study
☒	☐ Antibodies
☒	☐ Eukaryotic cell lines
☒	☐ Palaeontology and archaeology
☒	☐ Animals and other organisms
☒	☐ Clinical data
☒	☐ Dual use research of concern

Methods

n/a	Involved in the study
☒	☐ ChIP-seq
☒	☐ Flow cytometry
☒	☐ MRI-based neuroimaging
